# Design of a flexible component gathering algorithm for converting cell-based models to graph representations for use in evolutionary search

**DOI:** 10.1186/1471-2105-15-178

**Published:** 2014-06-10

**Authors:** Marianna Budnikova, Jeffrey W Habig, Daniel Lobo, Nicolas Cornia, Michael Levin, Tim Andersen

**Affiliations:** 1 Department of Computer Science, Boise State University, 1910 University Drive, Boise, ID 83725, USA; 2 Department of Biology and Tufts Center for Regeneration and Developmental Biology, Tufts University, 200 Boston Ave, Medford, MA 02155, USA

## Abstract

**Background:**

The ability of science to produce experimental data has outpaced the ability to effectively visualize and integrate the data into a conceptual framework that can further higher order understanding. Multidimensional and shape-based observational data of regenerative biology presents a particularly daunting challenge in this regard. Large amounts of data are available in regenerative biology, but little progress has been made in understanding how organisms such as planaria robustly achieve and maintain body form. An example of this kind of data can be found in a new repository (PlanformDB) that encodes descriptions of planaria experiments and morphological outcomes using a graph formalism.

**Results:**

We are developing a model discovery framework that uses a cell-based modeling platform combined with evolutionary search to automatically search for and identify plausible mechanisms for the biological behavior described in PlanformDB. To automate the evolutionary search we developed a way to compare the output of the modeling platform to the morphological descriptions stored in PlanformDB. We used a flexible connected component algorithm to create a graph representation of the virtual worm from the robust, cell-based simulation data. These graphs can then be validated and compared with target data from PlanformDB using the well-known graph-edit distance calculation, which provides a quantitative metric of similarity between graphs. The graph edit distance calculation was integrated into a fitness function that was able to guide automated searches for unbiased models of planarian regeneration. We present a cell-based model of planarian that can regenerate anatomical regions following bisection of the organism, and show that the automated model discovery framework is capable of searching for and finding models of planarian regeneration that match experimental data stored in PlanformDB.

**Conclusion:**

The work presented here, including our algorithm for converting cell-based models into graphs for comparison with data stored in an external data repository, has made feasible the automated development, training, and validation of computational models using morphology-based data. This work is part of an ongoing project to automate the search process, which will greatly expand our ability to identify, consider, and test biological mechanisms in the field of regenerative biology.

## Background

High-throughput technologies have led to an accumulation of large amounts of data that can be used to advance scientific inquiry given the appropriate tools. However, our inability to effectively visualize or conceptualize these data, particularly multidimensional data, is one of the factors preventing its integration into the scientific process. One of the promising means of using these data is to develop, train, and validate computational models, preferably those with interactive visual interfaces. Advances in computational modeling platforms are beginning to allow simulation of biological systems from the single cell biochemical level to more abstract multicellular environments, such as representative tissues, organs, or even organisms. These emerging computational tools are poised to put the power of bioinformatics and data interpretation back into the wet-bench biologists hands by automatically incorporating data from the aforementioned datasets with tools for visualization, experimentation, and data analysis.

Many high-throughput technologies collect large amounts of measurement data that are conducive to being stored in databases. For example, a database can easily house multi-scale gene expression data obtained from a single cell to a whole organism while also documenting the source and experimental methods associated with the data. Such repositories are well suited for data consisting of lists of gene and protein abundance, for example. However, new ontologies and formalisms are required for collecting and describing certain kinds of higher-order data. For instance, the outcome of experiments involving shape or morphology can be challenging to describe accurately, particularly in a way that others can search for or interpret computationally. This problem has been particularly challenging in areas of development and regeneration where a description of the organ, appendage, or organism is one of the key reported observations.

The planarian worm is a model organism in regenerative biology that perfectly illustrates the problem of storing shape-based experimental results in a formal database. These free-living flatworms have exceptional regenerative properties that have fascinated biologists for centuries [1]. They are able to regenerate aged, damaged, or lost tissues with the help of a large adult stem cell population [2]. Despite being complex organisms possessing bilateral symmetry, musculature, intestine, and a central nervous system including a true brain [3,4], fragments smaller than 1/200th of the adult size can remodel and regenerate an intact worm [5]. This astonishing regenerative ability has stimulated an effort to understand its underlying mechanisms [6], producing an extensive number of experiments based on amputations [4], drug-induced phenotypes [7,8], and RNAi gene-knockdowns [9-13]. However, despite these important efforts, we still lack a comprehensive model that can explain more than one or two aspects of planarian regeneration [14].

Recently, the Levin lab has developed a new tool (Planform) to aid in the assimilation of these data using a graph-based formalism to describe anatomy and morphology along with a new ontology for describing experimental manipulations and observations [15,16]. The flexible and extensible graph notation allows worm regions and organs to be described as nodes connected by linkages with associated angles and length parameters. Based on this approach, the Planform Database (PlanformDB) was designed and curated to include a complete description of the many planarian experiments and outcomes that exist throughout the literature. Such a resource does not only make it possible for scientists to search and compare worm morphologies, but it also provides an extractable resource for bioinformatics applications.

We are currently combining Planform, agent-based modeling, and an evolutionary search engine to develop an automated system for searching and validating computational models of regeneration. Agent-based modeling holds promise for studying the emergent behavior and complex interactions between signaling networks involved in directing regeneration, when multi-scale or multi-cellular systems are supported. To this end, we are using a modeling platform (CellSim) where the central agents are autonomous cells containing many of the biological primitives necessary for simulating living systems [17]. The current version of this software contains a number of useful features to support this endeavor, including a 3-D interface for visualization and tools for performing experimental manipulations within the client-server architecture. The process of developing, testing, and validating a complex model by hand can be a daunting task, particularly when many individual experimental outcomes are combined. To simplify this process, we have incorporated an evolutionary search engine that can automate this process using a genetic algorithm driven by appropriate fitness metrics that are informed by the Planform Database (PlanformDB). Our ultimate goal is for this integrated system to identify computational models that can account for many, if not all, of the available experimental outcomes related to planarian regeneration. We believe that this general approach holds the promise to spur biological discovery, develop novel insights into long-standing problems and biases, and elucidate previously unobserved biological behaviors.

This paper presents a novel agent-based planarian model capable of simulating basic biological behavior. The model is suitable for automated and varied experimental manipulations akin to those traditionally performed by wet-bench biologists and represented in the PlanformDB. This model includes a reaction network that responds to manipulations by initiating appropriate head and tail regeneration. Importantly, we describe an algorithm that allows translation of multicellular simulation output into a formal graph representation equivalent to that described by Lobo and colleagues [15,16]. This real-time translation is central to the automation of model discovery as it enables use of a fitness metric based upon a graph-edit distance calculation, which quantitatively compares simulation output and target morphologies stored in the PlanformDB. The combination of the model, translation algorithm, and fitness metric provide the basis for future automated model discovery in regeneration biology.

## Results

### Modeling a classic planaria regeneration experiment

**Figure 1:**
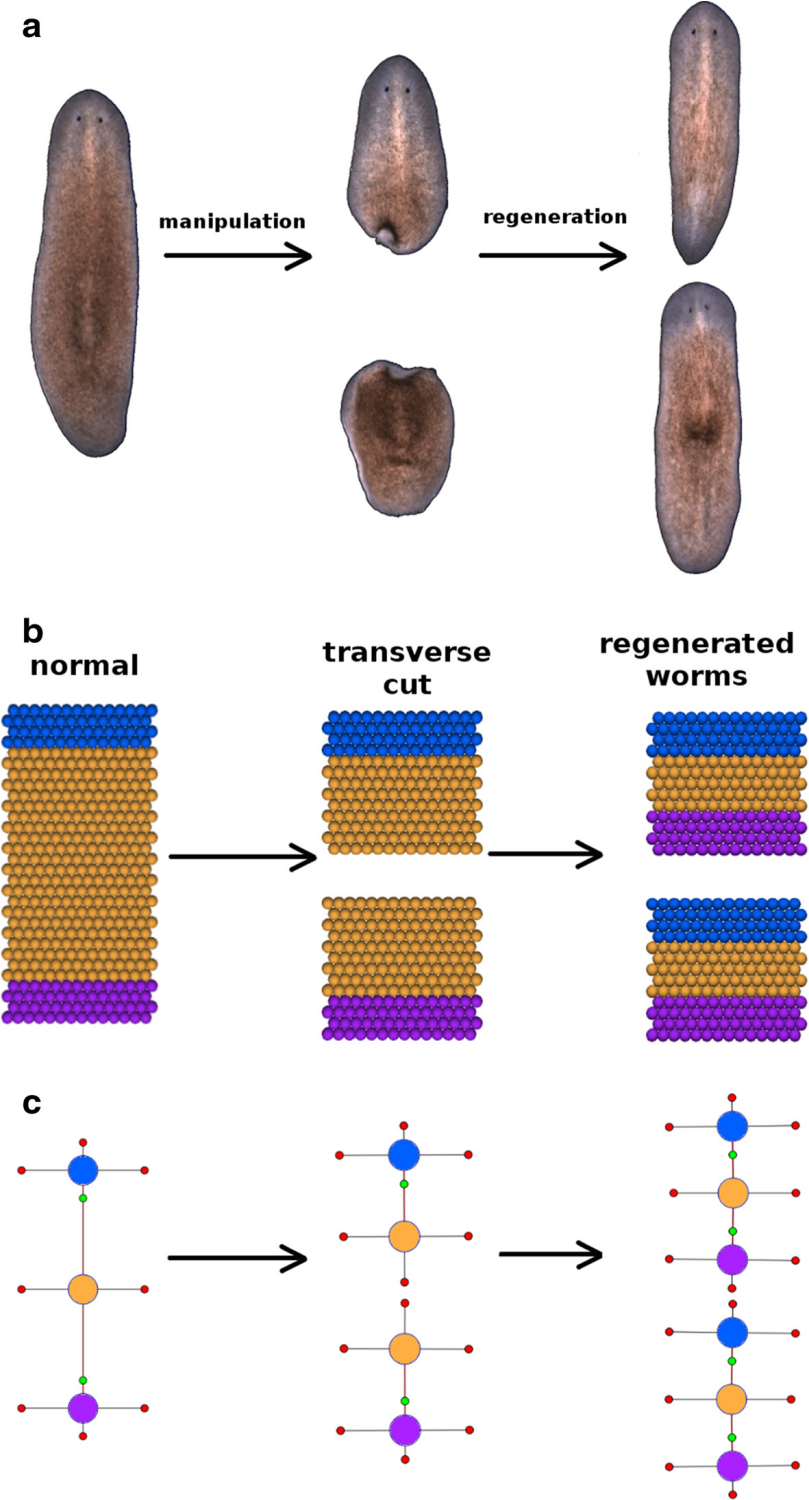
**This figure depicts a classic planaria regeneration experiment involving a transverse cut of an intact worm, followed by the regeneration products for each fragment.** The real experiment is shown in **(a)** along with the **(b)** simulation and **(c)** graph representations. In each case, the second panel represents the worms immediately following the cut, whereas the third panel depicts the regeneration outcome at a later time.

**Figure 2:**
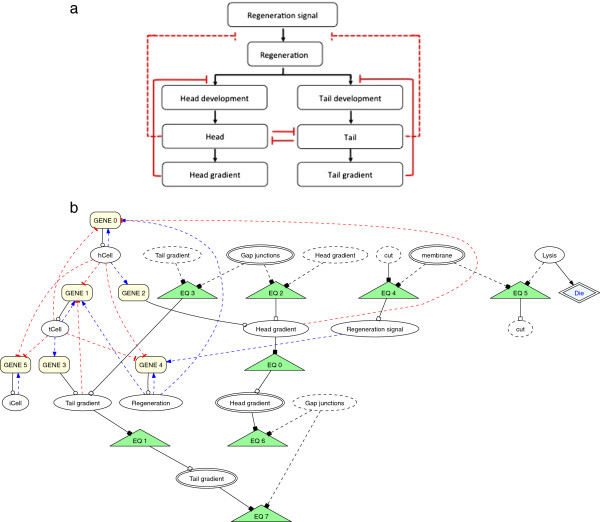
**General network scheme of the virtual planaria.** (a) Depiction of a regulatory model based upon a competitive development pathway initiated by a regeneration signal and repressed by active long-range morphogen gradients. (b) Representation of the principal components of the network as described in the Cellsim architecture. **(a)** Overview of general network scheme. **(b)** Regulatory network defined in Cellsim modeling platform.

As shown in the classic regeneration experiment presented in Figure 1a, when a worm is bisected laterally the resulting fragments will naturally lack a head or tail region. Normally, each fragment will regenerate into independent, intact worms with the appropriate shape and architecture over the course of roughly ten days. We sought to develop an agent-based representation of a planarian that could simulate these experiments. Such a model would (1) validate the chosen modeling platform (Cellsim, see Methods and [17]) for this project, (2) provide a working model for testing our translation algorithm and experimental manipulations, and (3) provide a starting network description to be used by individuals in the population of automated searches. The model developed was dependent upon competitive inhibition using a signaling mechanism consisting of long-range morphogen gradients emanating from existing head and tail regions. As the morphogen is subject to molecular decay and/or consumption in chemical reactions, its long-term presence is dependent upon the existence of its source (e.g. head or tail) in a given worm fragment. Thus, a regeneration signal can simultaneously trigger head and tail regeneration in response to a cut where the morphogens competitively inhibit their own developmental paths. For instance, the morphogen derived from head cells represses head regeneration in worm fragments possessing a head after a manipulation, whereas the lack of a tail will lead to decay of the tail morphogen and allow tail regeneration to proceed. A schematic of the network design used in these experiments is presented in Figure 2(a).

Our representation of this work included a simple architecture of 420 planar cells arranged as a rectangular abstraction of an intact worm (Figure 1b). The number of cells was chosen empirically to provide a robust system that could be reasonably manipulated by one or more simultaneous or sequential cuts. The implementation of our morphogen-based model was represented in Cellsim using a series of metabolic and transcription reactions (Figure 2(b)) where every cell was controlled autonomously by this same network. In response to a simulated cut, a *Regeneration signal* activates a *Regeneration* pathway, which simultaneously promotes head and tail development responses. The head and tail development pathways are constitutively repressed by the presence of a morphogen (i.e. *Head gradient* and *Tail gradient*) emanating from existing head or tail cells in the simulation and spread to neighboring cells through gap junctions. The morphogen gradients will disappear or be diminished following a transverse cut when the source (head or tail) is physically removed, causing one developmental pathway to be favored over the other. Furthermore, the head and tail resources repress each other to ensure a unique cell state is ultimately achieved.

At the start of a simulation, the head, trunk, and tail regions were defined by introducing one of three cell-state resources (head, *hCell*; trunk, *iCell*; tail, *tCell*) into each cell. Simulations were then run for approximately 200 steps to allow the network to reach homeostasis and provide sufficient time to develop long-range morphogen gradients. As shown in panel 1 of Figure 1b, worms consisted of head (blue) and tail (purple) regions separated by a trunk (orange). Next, a transverse cut was simulated by injecting a resource, *Lysis*, into a cross-section of cells located at or near the mid-line of the worm. The presence of *Lysis* results in a localized cell death response that results in separation of the initial worm into two worm fragments lacking either a head or tail (panel 2). Nearby cells respond to the *cut* by inducing a localized *Regeneration signal*, which in turn activates a cell’s *Regeneration* pathway. At this point, simulations consisting of two worm fragments were advanced another 200 steps prior to evaluating their emergent outcomes. The regulatory network parameters were optimized by hand for the network (Figure 2(b)), which resulted in proper regeneration of head and tail regions as shown in panel 2 of Figure 1b. For simplicity, these simulations did not include cell growth, division, or rearrangements, but these properties will be introduced in future studies.

These results showed that we could develop a simple model of planarian regeneration using a long-range morphogen gradient that could faithfully respond to at least simple manipulations. However, it was clear that hand-design and tuning a single model to represent the many experimental outcomes described in the literature would be a daunting task without computational automation. This challenge could be alleviated using an automated method of model creation and evaluation, such as performed by genetic algorithms [18]. These algorithms are based upon the principles of evolution where individuals in a population are generationally- modified through random genetic mutations and crossovers during reproduction. Individuals chosen to contribute to the offspring of the subsequent generation are selected, in part, based upon a fitness metric, which quantitatively defines how well each individual matches the characteristics of the target. This evolutionary search technique continues in an automated fashion until an individual matching the desired target (fitness value of 1.0) is generated.

### Graph formalism provides a convenient means of storing morphologies and comparing worms

The challenge of automating searches to identify possible planarian regeneration mechanisms was made more tractable by the database and formalism developed to describe wet-bench experiments and outcomes [15,16]. Within PlanformDB, worm morphologies are described using a graph-based formalism as part of a more general ontology for describing regeneration experiments. Briefly, a graph defines anatomic regions and organs as nodes where their size, spatial orientation, and connections are defined by parameters and linkages between adjoined nodes. For example, a simple description of the regions within a normal planarian consists of three connected nodes (head, trunk, and tail) as shown in Figure 1c. Although the formalism supports more complex descriptions of worms including organs, those aspects of worm anatomy were not considered in the current work. A particular experiment may include a description of the observed starting, intermediate, and ending morphologies of worms along with the physical or chemical manipulations performed in the laboratory. The database currently describes most, if not all, of the published planarian regeneration experiments for use by this and other projects.

In this study, we extended and adapted an existing genetic algorithm (CSGA) to fit our needs to model and evaluate planarian regeneration. One of the key adaptations was providing the CSGA access to the PlanformDB to facilitate simulation and fitness evaluation. However, the challenge of comparing our agent-based simulation output to a graph-based representation presented a significant challenge. In order to facilitate these comparisons, we chose to convert simulation results into a graph representation for a number of reasons, including increased flexibility as the CSGA could be extended to support additional modeling platforms as long as their output could also be translated into this graph formalism. More importantly, many methods currently exist for operating on, transforming, and comparing graphs which can be included as part of the fitness evaluation step of an automated evolutionary search [19-21]. Included in this repertoire are a number of algorithms suited for measuring similarity between two graphs [22]. Of these, the graph edit distance algorithm is the most flexible and powerful and was chosen here as it deals with structural errors and any type of graph node and edge labels [23,24].

The graph edit distance is defined as the minimum number of distortions required for transforming one graph into another. These distortions are referred to as graph edit operations, where each edit has a defined cost associated with it [23]. A particular sequence of edit operations is called an edit path, and the total cost of the edit path is the graph edit distance. Graphs that are similar to each other typically have small edit distances, whereas dissimilar graphs have large edit distances. The cost of each type of graph edit operation varies and is dependent upon the perceived severity of the operation. For example, the deletion of a node from a graph is generally viewed as having a higher cost than a node parameter change. Thus, the graph edit distance can be used as a quantitative similarity measure to compare and order individuals within a population, and thus serve as a metric within a fitness evaluation to guide the evolutionary search process.

### Design of a connected component analysis algorithm to convert cell simulation output into graph representations

**Figure 3:**
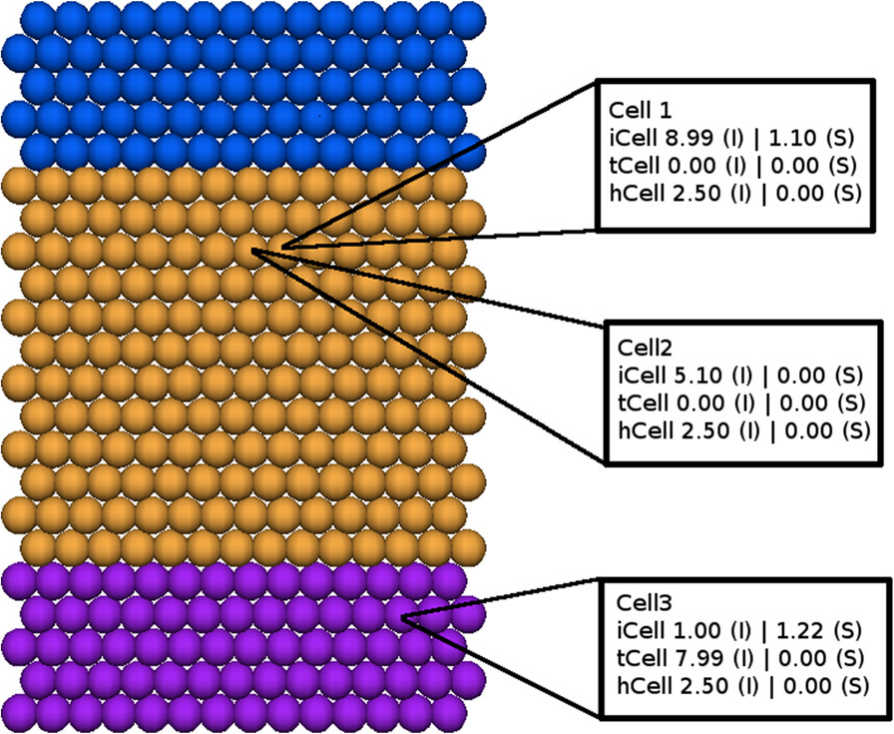
**The molecular concentration of resources varies between and within cells during a simulation.** The concentration of a particular resource is governed by spatial and environmental cues, such as signals from neighboring cells. Within a given cell the location of a given resource can be distributed between the internal compartment (e.g. cytosol) and the surface (e.g. membrane). The differentiate state of cells are color-coded to enable visual distinction of cells and the composition of a region: head (blue), trunk (yellow) and tail (purple). Concentrations of representative resources inside (I) and on the surface (S) are provided.

The worms in our simulation are composed of a collection of discrete cells rather than interconnected regions. Thus, the initial step in deriving a graph-based representation is to translate cells within a simulation snapshot into discrete regions (e.g. head, trunk, or tail), and determine how they are interconnected. To this end, we designed and implemented an algorithm based upon connected component analysis similar to methods used in computer vision and document analysis [25].A simulation is processed as a series of discrete steps where each step consists of a complete description of cellular locations and their individual resources (snapshot). The snapshot associated with each step can be independently analyzed by the algorithm to identify cell states and identify regions over time, or a single endpoint can be examined. The algorithm first iterates through all the cells in a snapshot and assigns each cell a region type (e.g. head, trunk, or tail). In general, the assignment of a cell type could be complex as there are many different components (e.g. proteins/resources or neighboring cell interactions) associated with each cell. In our case, we decided to simply define each cell’s state based upon the molecular concentrations of three resources, which we labelled hCell (head), iCell (trunk), and tCell (tail). Our algorithm assigns a region type to each cell based upon the highest total concentration of these indicator resources. For example, a cell is assigned a head state if its concentration of hCell is greater than iCell and tCell. In the modeling platform, transcriptional noise and cellular autonomy result in cells with varying concentrations of resources, even those located near each other (Figure 3). Since a resource may be found on the cell surface (S) or internally (I), the algorithm was designed with the flexibility to allow the user to define whether to consider the total or localized concentrations of resources. We used total cellular concentration of each of the three indicator resources to determine a cell’s state in this work.

Once each cell’s state has been determined, the algorithm identifies regions based upon spatially cohesive cells sharing the same state using connected component analysis. All cells located adjacent to cells containing the same state are considered part of the same region, where the outermost cells define the region border. The connected component analysis algorithm (Algorithm 1) initiates with a call to the ProcessConnectedComponents function providing a simulation snapshot as a parameter. The ProcessConnectedComponents function cycles through all cells and calls the GatherConnected function for each unassigned/marked cell. The GatherConnected function recursively collects and marks all other cells in the snapshot that belong to the same spatially cohesive region as the starting cell. A cell is defined to be in the same spatially cohesive region as the starting cell if it is of the same type as the starting cell and is either connected to the starting cell or to some other cell already determined to be in the starting cell’s region. Two cells are considered to be connected if the Euclidean distance between them is below a user-specified threshold (discussed in section ‘A cell connectivity distance threshold effects region determination’). Additionally, if two cells are close enough to each other to be considered connected, but are assigned to different regions because they are of different types, those cells are identified as border cells. Border cells are used to determine which regions are linked to each other. 

Once each cell in the snapshot is assigned to a specific region, the algorithm determines the number of neighboring regions using the border cells found during the recursive process and establishes links between nodes where regions are considered linked if their border cells are adjacent to each other. Other necessary parameters for a complete graph representation include the distance between the connected regions’ centers (length of link), orientation with respect to each other (angle of the link relative to the x-axis), and the border between the two regions (location along the link where the two regions meet). The center of a region is calculated by averaging the spatial centers of every cell within a particular region. The Euclidian distance between these points of neighboring regions is used to define the length and orientation of each link. Finally, the graph component parameter defining the borders of regions in each direction is calculated from the location of the most distantly located cell in a specific direction. The number of parameters for a region depends on the number of links it has with other regions. Figures 1(b to c) show examples of simulation morphologies that have been converted to a graph formalism using this algorithm.

### Simulation snapshots are converted to well-ordered graphs using our conversion and graph-edit distance algorithms

**Figure 4:**
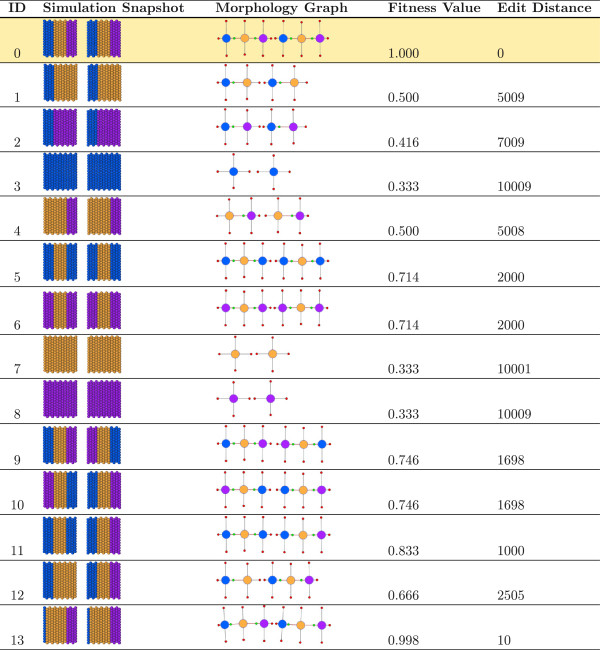
Single cut morphology experiment results for simulation snapshot to graph conversion and graph edit distance comparison.

During an evolutionary search, large numbers of unique individuals are generated and must be evaluated against the target individual encoded in the database. Thus, we sought to evaluate our conversion algorithm manually to ensure the graph representations were intuitive and to evaluate the use of the graph edit distance metric for ordering individuals in the population. To this end, we generated a number of worms with distinct morphologies by hand using the modeling platform and converted their snapshots into graph representations using our algorithm. The simplest individuals that can be represented by the simulation platform include worms with discrete regions, whereas more complicated morphologies consisting of regions contained within other regions could also exist. Just considering the basic morphologies, the number of individuals that can be formed and the search space for the genetic algorithm are infinite, and therefore our algorithm was tested using simple individuals before considering more complicated morphologies.As shown in Figure 4, we generated a series of distinct worms (ID 1-13) for comparison with a desired target (ID 0). In each case, the worm representations included two fragments to simulate the state of worms following a single transverse cut. Each worm was generated by injection of the appropriate cell-state resource (i.e. hCell, tCell and iCell) to generate the desired regions within the worm fragments, resulting in different permutations of head, tail and trunk regions. Every test morphology was converted to a graph (Figure 4, Morphology Graph) using our conversion algorithm. We did not find discordance between the graphs generated by the conversion algorithm and those expected upon visual inspection of the simulation output. Thus, the algorithm was working as expected on these simple morphologies.During an evolutionary search, the genetic algorithm needs to compare individuals to the target and reward those individuals with morphologies most similar to the target individual as their offspring are more likely to possess the a reaction network capable of proper regeneration. The genetic algorithm thus assigns fitness values based upon how similar the individual is to the target, with more similar individuals getting higher fitness values. A fitness value of 1.0 is awarded to an individual with a perfect match to the target and is the ultimate goal of a search. Thus, we calculated the graph edit distance between each test individual and the target and converted those values into a fitness value (Figure 4).

The graph edit distance penalties used in our algorithm and this manuscript are described in Table 1, but can be modified by the user. The penalties are most severe when differences exist between region numbers and connectivity than for region size and linkage parameters. While the optimization of the graph edit costs is beyond the scope of this paper, we will explore methods for automated optimization of the graph edit costs in future work.The target individual, when compared to itself, yielded a value of 0.0, because when two individuals are identical, the distance between them measured by the graph edit distance algorithm is 0. Using Equation 1, the distance of 0.0 translates to a fitness value of 1.0, which in a genetic algorithm search would indicate the target morphology has been found. Morphology 13 in Figure 4 is a slight variation of the target morphology, where its heads are several cell layers thinner than the heads of the target, and as expected, has the next best fitness value (0.998). In general, high fitness values for morphologies such as number 13 are expected as their regions are connected and oriented the same as the target. When compared to the target, morphologies that consist of three regions in each worm fragment received higher fitness values than morphologies having one or two regions. For example, morphology 6 was rated higher than morphology 4. Again, this is because the graph edit distance costs included a much larger penalty for the deletion of a region than with a change to the type of region.

**Table 1:** This table presents the graph edit costs used for graph edit distance calculations in this manuscript

**Operation**	**Cost**
Insert/delete region	1500
Change region type	1000
Change region parameter	0.1 per unit changed
Insert/delete link	1000
Change link distance	0.1 per unit moved
Change link angle	0–100
Change link angle > 90 penalty	750

**Figure 5:**
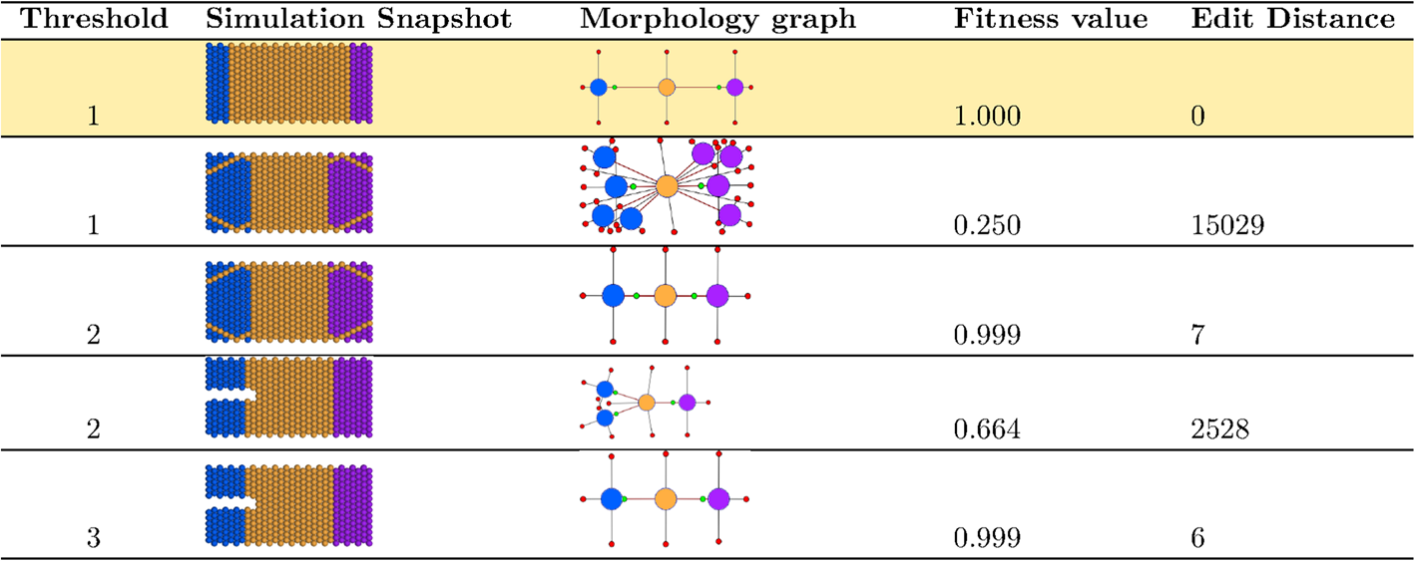
Threshold value influence on snapshot to graph conversion algorithm.

Conversion of a simulation snapshot into the graph is an *O*(*N*^2^) algorithm where *N* is the number of cells in an individual. The wild type morphology had 420 cells, but since the transverse cut removed four rows of cells, the morphologies used in this experiment consisted of 364 cells. The conversion algorithm ran in less than 1.3 seconds for every morphology in Figure 5. It is well-known that the run time of the graph edit distance calculation grows exponentially with the size of the graphs (number of nodes, or in our case to the number of regions in the two morphologies) [23]. However, since the number of regions in each morphology tested was at most 6, the graph distance algorithm finished in less than 1 millisecond for all morphologies.

### A cell connectivity distance threshold effects region determination

A comparison of more complicated morphologies highlighted the need for a flexible distance threshold in the component gathering algorithm. Since the cells in our simulation have radii of 0.5 units, the Euclidian distance between two adjacent cells can be as low as one. However, using a very rigid measure for identifying neighboring cells and determining the borders of regions can have dramatic effects on the graph conversion. For example, consider the morphology of the second individual shown in Figure 5. In this individual thin lines of trunk cells dissect the head and tail regions into a number of potentially distinct heads and tails if the borders are considered rigidly. Comparison of this individual with the target results in a very high graph edit distance due to the cost associated with having multiple heads. However, in the context of a evolutionary search, this individual may be very close to producing the target morphology.

A flexible threshold parameter was introduced to reduce the rigidity of region definitions, which allowed neighboring regions separated by thin regions to be merged in the final graph representation. Increasing the threshold value reduces the stringency by increasing the search distance between cells for neighbors with the same state. Thus, in the example just discussed, increasing the threshold value allowed the multiple head regions to be lumped into a single head region. The graph edit distance of this worm is much lower resulting in a fitness value close to one.A second example highlighting the importance of this parameter to component gathering is presented at the bottom of Figure 5. This worm represents a classic experiment that involves bifurcating the head region into two fully-developed heads. These two heads are separated physically and should be classified as two-headed. A threshold parameter of less than three results in the desired graph conversion in our algorithm, whereas the larger value results in a worm with a single head.

These two examples show the necessity of a flexible parameter for determining local regions during a GA run. In the first case, a low threshold was shown to penalize a morphology that was very similar to the target, whereas a high threshold inappropriately favored a morphology containing a physical gap between head regions. An optimal threshold will depend upon the modeling platform and project, but in this work and from an evolutionary perspective a threshold of two was optimal.

### Validation of component gathering and graph edit distance during evolutionary search

**Figure 6:**
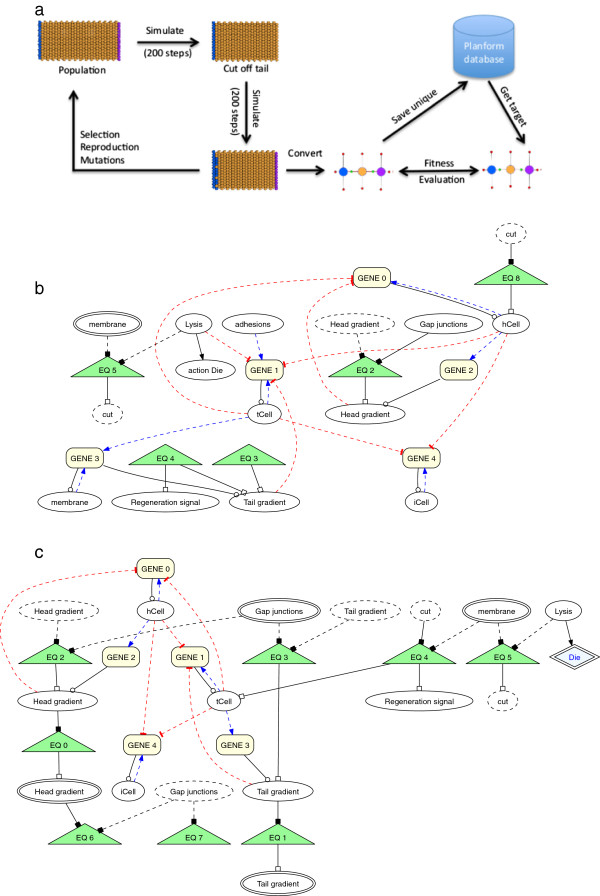
**An evolutionary search experiment where simulated individuals are evaluated against a target morphology extracted from the PlanformDB.** The simulation output is converted to a graph using our component gathering analysis algorithm followed by a comparison using the graph edit distance fitness function. The process is repeated with new individuals following selection, reproduction, and mutation operations until a suitable solution is identified. Solutions from simple searches are presented following removal of the anterior (b) or posterior (c), respectively. **(a)** Genetic Algorithm (GA) experimental flow. **(b)** Representative GA head regeneration solution network. **(c)** Representative GA tail regeneration solution network.

As our ultimate goal is the generation of an automated model discovery tool, we tested the utility of our conversion algorithm and the graph edit distance as a fitness metric as part of an evolutionary search for a target described in the PlanformDB. As shown in Figure 6a, we selected an experiment from the database where either the anterior or posterior end of an intact worm was removed. Functionality was added to the GA to interact directly with the PlanformDB so that it could extract the target individual as a graph representation for comparison. The target individual for this experiment was a single normal worm consisting of head, trunk, and tail regions connected in that order (see graph in Figure 6a).

The starting population of individuals in the GA search contained a version of the hand-designed model description shown in Figure 2 after it was purposely modified to become non-functional. The *Regeneration*, *Head development*, and *Tail development* resources were removed from the regulatory network, thus preventing proper regeneration (data not shown). We were interested in whether the GA could find solutions that properly regenerated the tail region using our conversion algorithm and a fitness metric based solely on the graph edit distance calculation. Since the GA is designed to evaluate fitness values in the range of 0.0 to 1.0, we converted the graph edit value as shown in Equation 1. Initially, we used the simple inverse function of 1/(graph edit distance) to obtain the GA fitness values. However, the fitness function values in such cases tended to be very small even for relatively similar graphs due to sensitivity caused by the large edit penalties in Table 1. To reduce the sensitivity to the large edit distance penalties, we introduced a constant (5000) to our equation after empirically testing values between 1000 and 10000. Constants below 1000 were excluded as they yielded very small fitness values, while constants above 10000 were excluded on the basis of generating high fitness values for individuals with morphologies very dissimilar to the target. Although other numbers in this range could have sufficed, the 5000 value maximized the differences between fitness values and thus was used in this work.

(1) $$\text{fitness}=\frac{5000}{\left(\text{distance}+5000\right)}$$

Our searches included populations of only 30 individuals, but much larger populations are feasible. In order to generate variability in the individuals, a set of mutation and crossover parameters were introduced and applied to each new generation of offspring. These operators and parameters were hand designed for this experiment, but parameter definition will eventually become automated. Searches were performed for individuals with proper regeneration of head or tail following removal of the anterior or posterior regions, respectively. During the evolutionary search, the GA pauses each simulation at a predefined step (e.g. 200 in this experiment) and requests a simulated experiment be performed (e.g. cutting off the head or tail). Each individual simulation is resumed and continues until the GA requests a snapshot to evaluate (e.g. step 400). At this point, the simulation snapshot is used to create a graph representation using our component gathering algorithm, which is then compared to the target individual from the PlanformDB to determine the individual’s fitness value. High scoring individuals were chosen for reproduction to generate the next generation of individuals which were independently mutated to increase variability in the population.The GA was successful in identifying individuals with fitness values very close to the target value of 1.0. Two such regulatory networks are shown in Figure 6. The network shown in Figure 6(b) is a representative evolved solution when selecting for a worm that properly regenerates head after removal of the anterior region of an intact worm. Similarly, the network in Figure 6(c) comes from a representative worm that was evolved to regenerate a tail following removal of the posterior end. There are noticeable similarities between the two solutions. In both solutions, a direct connection was made between the cut response to regeneration of the missing region, head or tail, respectively. Although each search found a solution to the experiment at hand, the solutions were limited in their flexibility to respond to other permutations. As expected, neither network was capable of solving the reverse problem as their was no selective pressure in the evolutionary search. Nonetheless, these results show that our evolutionary search process is capable of finding solutions using our connected component analysis to convert cell-based individuals into graphs, which are compared with the appropriate target extracted from the PlanformDB using the graph edit difference evaluation metric.

The inflexible network solutions emphasize the importance of searching for solutions using rigorous fitness criteria and why an automated approach is necessary. Future experiments will target networks that are capable of handling both anterior and posterior ablations. Simulation snapshots contain a detailed description of the current state of the simulation, including a list of all cells, their location, shape, genomes, metabolic equations, environmental conditions, and the concentration of all resources. Thus, snapshots provide much richer information about the cells and individuals than the graph formalism and will provide opportunities to develop additional fitness metrics to complement graph edit distance in the future.

## Discussion and conclusion

One of the challenges in the biological sciences is the development of new methods for data visualization and integration to provide informative and predictive insight into the scientific process. Computer models hold great promise in this area, but often involve significant human interaction and time. In this study, we laid the foundation for a system of automated model discovery and development that incorporates shape-based experimental data from a repository of documented experiments. Graphs are a powerful and convenient means of describing morphological data. Using comparison methods, such as the graph difference evaluation, one can easily search such a database for results that are similar or identical. We showed that the utility of the graph difference evaluation could be further extended as a fitness evaluation metric during evolutionary search. This method was combined with a cell-based modeling platform to model basic regeneration of the planarian flatworm. Agent-based models are particularly amenable to this approach as they are tractable to simulated experimental manipulations combined with fully emergent outcomes. The ability to automate these behaviors fits nicely into an automated discovery system that can be driven by a genetic algorithm search engine. Furthermore, simulators that include robust visualization capabilities make it very convenient for the scientist to evaluate or experiment on a set of search results.

Planarian worms provide an excellent model system for developing such an automated search process due to the plethora of experimental data in the literature, and now available in a curated database (PlanformDB). However, the principles inherent in this design are extensible to any system where shape is an integral component of the observable outcomes. That said, developing model discovery systems that automatically incorporate experimental data is a general and attainable goal that is not limited to systems dependent upon morphological data.The challenge of describing phenotypic outcomes based upon morphological characteristics is challenging for biological systems and cell-based computational models alike. We showed that converting cell-based simulation output into graphs can be achieved using a component gathering algorithm that identifies regions and their juxtaposition to each other and converts them into nodes joined by linkages. The resulting graphs can be stored to a database and easily reconstructed later and/or compared with other graphs using algorithms such as the graph edit distance. These comparisons and the resulting metric were incorporated into an evolutionary search where the genetic algorithm retrieved its target morphology from a data repository of experimental outcomes and used the graph edit distance as a fitness metric to drive development. Using a small population size of 30 individuals, our mutation and crossover frequencies were sufficient to generate a solution state in as few as 19 generations for the experiments shown in Figure 6(a).

The method for converting a simulation snapshot to a graph formulation works well, and the converted morphologies reflected shape and positions of body regions relative to each other in space. The graph-based fitness function thus accurately distinguishes the shapes of different morphologies, but does have difficulty predicting which morphology will more likely regenerate into the target morphology. To improve the effectiveness of our graph-based fitness function, future work will seek to automate the optimization of the graph edit costs to not only reflect the shapes of individuals, but also to favor morphologies that are more likely to regenerate into the target individual. The optimization of graph edit costs needs to be automated since tweaking graph edit costs allows one to achieve a more accurate graph comparison for some cases, but there is no perfect parameter assignment that covers all cases, and so it should be chosen depending on the needs and design of the experiment.

Future work will also develop additional morphological based fitness functions to act side by side with the graph edit distance fitness function. This will allow researchers to choose from among multiple morphological fitness operators, possibly combining the output of several evaluators instead of assigning a fitness value based on one fitness function evaluation.

## Methods

### Cell-based modeling platform

Cellsim is an agent-based modeling platform whose principle agents are cellular in nature. These cells and their behaviors are rooted in biology as the environment, interactions, metabolism, and signaling networks are designed based upon biological primitives. Cells are capable of proliferating, growing, dividing, dying, and regulating metabolic and genetic networks in response to changes in their local environment, including cell interactions and signaling. As a result, cells have emergent properties as they are autonomous, evolvable, exhibit inheritance, and are contingent upon their neighbors. Another important feature of this system is its ability to be automated and manipulated using a genetic algorithm search engine [17].

The versatile genetic algorithm associated with Cellsim was expanded as part of this work and includes many parameters to customize the common elements of a genetic algorithm, such as number of crossovers, mutation rates, selection criteria, and population size.

### Graph edit distance algorithm

Formally, to transform the morphology graph *g*_1_ into graph *g*_2_, a sequence of operations (the edit path) must be performed [23,24]. The edit distance between two graphs is defined as the minimum cost edit path that transforms graph *g*_1_ into graph *g*_2_ as represented in Equation 2.

(2) $$d(g_{1},g_{2})=\text{mi}n_{\left(e_{1},\mathrm{..}e_{k})\in P(g_{1},g_{2}\right)}\overset{k}{\underset{i=1}{\sum }}c(e_{i}),$$

where *P*(*g*_1_,*g*_2_) is the set of edit paths that transform graph *g*_1_ into *g*_2_, *c* is the edit cost function and *e*_*i*_ denotes an edit operation. Generally speaking, determining the graph edit distance requires that we examine the set of paths that transform *g*_1_ to *g*_2_ and calculate the path costs for each. This is non-trivial but can be achieved in an optimally efficient manner using the A* best-first search algorithm [26].

The graph edit distance calculation has been adapted for comparing planaria graph representations. A list of edit operations and an example of the corresponding costs used in this paper are given in Table 1.

For simplicity, we are working with worms whose graph representations are devoid of organs for this analysis, but will include them in later experiments.

The minimal path cost between two graphs *g*_1_ and *g*_2_ is found using the A* search algorithm. The possible edit paths can be viewed as forming a tree, where the edges of the tree are individual edit operations and the nodes are graphs. The inner nodes of the tree correspond to partially edited graphs, and the leaf nodes represent complete edit paths, all of which terminate at the target graph. The search for the minimal edit path starts with one of the graphs, say *g*_1_, as the root of the tree, and defines the possible branches from this node to be all possible single edit operations that could be applied to the original graph *g*_1_. At each step of the A* search, the algorithm expands (explores) the branch of the tree that leads to a node on the search frontier that has the minimum estimated total path cost. The minimum estimated total path cost is derived from the sum of the cost of the edits required to reach the node being expanded from the root, plus an estimate of the total cost of the edits required to reach the goal state (graph *g*_2_) from the node being expanded. The algorithm terminates the first time it expands the goal state, as the path that it finds at this point is guaranteed to be optimal.

## Competing interests

Tim Andersen and Jeff Habig are former employees of Crowley Davis Research who developed the Cellsim platform, but are no longer associated with the company in any way and declare no financial interest or conflict of interest for themselves or any other authors. Crowley Davis Research is not directly involved in this project.

## Authors’ contributions

Component gathering design, development, and analysis (MB, NC, and TA). Modeling, network design, and genetic algorithm searches (JH). Experimental design and interpretation (JH, TA, and ML). Graph-edit distance evaluation and live planarian experiments (DL). Manuscript preparation (MB, JH, and TA). All authors read and approved the final manuscript.
